# Balancing Pragmatism and Sustainability: A Case Study of an Interorganisational Network to Improve Integrated Care for the Elderly

**DOI:** 10.5334/ijic.5635

**Published:** 2021-09-27

**Authors:** Anna Hallberg, Ulrika Winblad, Mio Fredriksson

**Affiliations:** 1 Health Services Research, Department of Public Health & Caring Sciences, Uppsala University, Sweden

**Keywords:** integrated care, interorganisational collaboration, networks, leadership, Sweden

## Abstract

**Introduction:**

Networks constitute a promising strategy for interorganisational collaboration, but may fail due to network tensions. By investigating the activities and internal dynamics of a voluntary meso-level network operating in the intersection of health and social care, this study aims to enhance the understanding of the relationship between pragmatism and sustainability and the role network governance plays in this respect.

**Methods:**

In this descriptive case study, 2–3 researchers observed 3 three-hour long network meetings during the course of a year, and four complementary interviews were performed. Data were analysed based on the literature on network functioning and effectiveness.

**Results:**

Pragmatism (a focus on ‘getting things done’) was more emphasised than sustainability although the network meetings also contained elements of relationship- and trust-building. The network leadership (a Network Administrative Organisation, NAO) created structure and concretized the participants’ ideas while remaining flexible and perceptive, and also carried out tasks which would otherwise not have been performed.

**Discussion:**

The emphasis on pragmatism did not seem to influence sustainability negatively which has been pointed out as a potential risk in previous literature. Rather, the focus on pragmatism reinforced sustainability in a way that is similar to what has been described in prior research as a “trust-building loop” and discussed further in terms of a “perception of progress” mechanism. However, it was unclear what future the voluntary network would have without the NAO.

**Conclusion:**

Network governance is instrumental to success, and should be carefully considered when initiating interorganisational network initiatives for integrated care.

## Introduction

Health and social care for the elderly is often fragmented [1] and integration can potentially enhance quality, reduce costs and lead to increased satisfaction [2,3]. Achieving more integrated care requires not only interprofessional collaboration – the most common approach [4] – but also *interorganisational* collaboration [5]. *Networks*, defined as “groups of three or more legally autonomous organisations that work together to achieve not only their own goals but also a collective goal” [6, p. 231], are one way to improve interorganisational collaboration [7]. Although studies suggest that networks can enhance performance and have positive outcomes, the evidence is uncertain [8]. There is furthermore limited insight into the circumstances under which networks for achieving integrated care are successful, and a substantial number of these networks fail [5].

A common theme in the broader network and partnership working literature is that different core values and principles, for example accountability, pragmatism, flexibility and sustainability, must be balanced [9,10]. Their presence may vary or shift over time, and they may sometimes be in conflict. The reason for why networks function or fail may thus be sought in the relationship between different network principles.

In this article, we focus on the relationship between the principles of *pragmatism* (e.g. getting things done, meeting targets) and *sustainability* (e.g. fostering participation, building consensus). This may also be expressed in terms of network results versus network relations [11], or network outcomes versus network processes [12]. These two principles have been highlighted as important in recent literature investigating the effectiveness of networks. Newman [9] exemplifies how a pragmatic focus on short-term goals may limit sustainability by side-lining inclusive and participative activities which build network sustainability. However, the literature also indicates that trust among network members may be strengthened by the joint achievement of common goals [13,14]. This suggests that a pragmatic approach might not limit sustainability, and calls for a better understanding of how the principles are related in different types of networks. For example, it has been suggested that the occurrence of potential tensions may decrease over time, but that more mature networks need to be studied [11].

Furthermore, how the network is governed or managed (also discussed as network leadership [14]) may be a key factor in mitigating potential tensions and achieving balance between network principles [6]. At the same time, Klaster et al., [11] illustrates that a coordinating versus a leading governance style can affect potential network tensions differently, suggesting that a more nuanced understanding of network governance might be needed. There is also research suggesting that network governance through different types of broker organisations can weaken the collaboration by limiting sustainability through their gradual assumption of the network partners’ tasks, and by taking decisions in their place [15].

To further study how these principles are related in different types of networks, we investigate the activities of a network operating at the meso-level (while most strategies for integrated care target the clinical level) in the intersection of health and social care [4]. The network, based on voluntary participation, is governed by a Network Administrative Organisation (NAO) and has a twofold purpose, aiming both to achieve pragmatic outcomes and to foster a sustainable collaborative arena. By investigating its activities and internal dynamics, our aim is to enhance the understanding of the relationship between pragmatism and sustainability and the role network governance plays in this respect. We thus contribute to the understanding of mechanisms underlying the functioning and effectiveness of interorganisational collaborations in health and social care, which can be used to further refine theory about interorganisational collaboration and about barriers to integrated care, in particular at the meso-level [7,14]. This is needed since many networks struggle to balance between competing values and principles and to achieve their goals.

## Theory: the principles of pragmatism and sustainability and their relationship

As previously mentioned, the principles of pragmatism and sustainability may not always be compatible. For example, if too much pressure is put on the network actors to achieve quantifiable or short-term results, inclusive and participative activities may be side-lined, jeopardizing long-term network objectives [11]. Similarly, it has been suggested that striving towards network efficiency may conflict with inclusiveness, since inclusive decision-making is a more time consuming and resource intensive process. It has also been highlighted the importance in networks of being responsive to external demands [6], which might sometimes come in the form of more short-term quantifiable goals related to efficiency, suggesting that difficult trade-offs between the two principles can occur.

Huxham & Vangen [13] suggest that *trust*, which is important for realizing more ambitious goals in the long-run (sustainability), can be built by initially achieving less far-reaching common goals (pragmatism). This more complex interdependence between the two principles, described as a “trust building loop”, has been highlighted in recent research where the authors elaborate further on the link between trust and perception of progress. Unambitious or overambitious aims, respectively, can affect the perception of progress among network members so that trust is either enhanced or reduced [14].

From this, it is clear that the relation between sustainability and pragmatism in networks can vary, and there is an increasing focus on the circumstances that promote tension or balance [15]. An important contributing factor is network governance, sometimes divided into shared-participant (governed by organisation members), lead organisation (one member/organisation takes the lead) and NAO-governed [6]. Research shows that management in a collaborative setting means working with a combination of congruent and diverse goals, and requires an acceptance of potential tensions [16]. Lately, NAOs have been further investigated. It has been stated that in relation to the efficiency/inclusiveness tension described above “an NAO mode of governance” is likely to provide balance between the two by allowing for structured and representative participation while also providing administrative support [6]. It has also been suggested however that an NAO, by virtue of its leadership style being more or less coordinating or leading, can affect potential network tensions in different ways [11] and that NAOs and other broker organisations may sometimes hamper rather than support collaborations [15].

For analytical purposes, i.e., in order to capture the occurrence of and relation between pragmatism and sustainability in the studied network, the most essential components of the two principles, as described by Newman [9], are summarized in ***Table 1***. Somewhat simplistically, a focus on *pragmatism* may cause a marginalization of differences between network participants in the search for a group that can pursue a common agenda, excluding less powerful participants. In this case, leadership tends to be directive rather than inclusive and decision-making is fast and informal. There is little investment in trust-building, which may eventually act as a barrier to sustainability. In contrast, the route towards *sustainability* focuses on empowerment and participation, and participants taking responsibility for actions and outcomes [9]. Klaster et al. [11] talk about establishing enduring network relationships including aspects such as trust, communication, and commitment to a common purpose. Furthermore, working towards sustainability implies a tendency to include different interests and views, and acknowledging differences is seen as an essential part of the process. Leadership may be described as inclusive and empowering.

**Table 1:** Components related to the principles of pragmatism and sustainability.


	PRAGMATISM	SUSTAINABILITY

Focus of network activities	Joint activity around specified objectives, often short-term goals Goal attainment (individual/organizational) Getting things done	Participation Building consensus/trust Empowering participants to take responsibility for actions and outcomes, capacity building
Reason for collaboration	External requirements and obligations Response to opportunities	Embedding of network for long-term development Commitment to common purpose
Leadership	Marginalise differences to get things done Directive style Fast and informal decision-making	Acknowledge differences and include different interests and views Coordinating style Inclusive and empowering


## Methods

### The case

In this descriptive case study, we investigate a network that had at the time of the study in 2019 been operating for over five years in the southern part of a Swedish region. It was initiated in 2015 by a local R&D centre that is co-owned by the region (responsible for health care) and several constituent municipalities (responsible for elder care).

The overall purpose of the network is to constitute a sustainable collaborative arena enabling different health and social care actors to share experiences, knowledge, and inspire concrete and practical development work [15]. The network is open to representatives from eldercare, primary care, and specialist care administered by municipalities, the region, and private providers. An explicit goal is to include managers with some level of operational capacity [17]. Today, the network consists of representatives with different positions within their respective organisations, including municipal and hospital operational managers, but also professionals with coordinating functions and municipal case officers.

The activities of the network consist of four regular meetings per year where the voluntary participants gather for 3–4 hours. In between these meetings, there are additional meetings in working groups tied to specific projects. The R&D centre employs two meeting leaders, giving them the role of a so-called NAO, but no other funding exists. The NAO leaders provide an agenda before each meeting and send out detailed notes after each meeting. At present, the mailing list consists of around 40 people, with about 20 participants partaking at a typical meeting.

### Data collection and analysis

This study draws primarily on observations carried out during three regular network meetings. 2–3 researchers per meeting took field notes as “complete observers” [18], resulting in about 21 hours of observation. Observations were guided by two pilot observations as well as a checklist developed from theory, summarizing important aspects of network functioning [19]. To validate and complement the observations, four semi-structured interviews were also conducted with four network members from different organisations that had participated at least once during the time of the observations in 2019, lasting 30–45 minutes each. The interviews captured aspects of network dynamics that were not possible to observe, such as participants’ motivations for and experience of, participating in the network.

In the analysis, field notes were first organized by compiling text segments from all three researchers about the same part of the meeting, making it easier to compare observations. Two of the researchers then carried out a first analysis, focusing on the character of network activities and processes, as well as the leadership. This work guided the development of the theoretical specification and formulation of the interview guide. In a second step, the field notes and the interviews were assessed more comprehensively through a thematic analysis [20] using code words for pragmatism and sustainability respectively. By analysing the extent to which, and in what way, the two approaches outlined in ***Table 1*** appeared during the meetings, conclusions regarding their relationship, and the role of the NAO in this process, could be drawn.

### Ethical statement

The study was approved by the Swedish authority for ethical review (no. 2019-02520). Both the participants in the observations and the interviewees provided written informed consent to participate. They were informed in writing and orally about the purpose of the study and that participation was voluntary.

## Results and analysis

### Pragmatism: creating products

The analysis of the observations and interviews disclosed that both principles were present in the network, but that pragmatism was more emphasised. A considerable amount of time at the network meetings was put into the planning and organizing of projects, and reporting on how things had progressed since the last meeting (the work continued between meetings in smaller working groups). It appeared important to the participants that the meetings were about *getting things done*, and the NAO leaders often pointed out that the meetings were not the type where people “just sit and talk”, but that they should lead to *joint activity*. In the interviews, it was expressed that the focus on getting things done made people keep coming to meetings:

“You feel that you do something /…/ Otherwise it feels meaningless if you only sit in a group.” (Interview 4)

Because a new law (2017:612) focusing on collaboration for elderly when discharged from hospital was introduced in 2018 discussions on how to implement the law locally, and problems associated with it, was a recurrent theme at the meetings. According to the law, the discharge process should follow a certain routine (including a joint care-planning meeting between regional and municipal actors, a so-called Coordinated Individual Plan or SIP), and one of the network’s most featured projects was to translate this routine into local work procedures. Other recurrent projects were the planning of a workshop where staff from different organisations could meet, a mapping of care transitions for the elderly, and a mapping of support functions for elderly with poor mental health.

Some projects were *responses to opportunities*, for example a project initiated after external funding from a state agency was received, and in some cases, the observations suggested that the projects pursued by the network were those deemed possible to carry out, rather than those the network participants would ideally see executed. The participants’ potential for engagement was found to be a major obstacle for taking on more ambitious tasks. In the interviews, it was pointed out that some of the projects required too much time from the participants, making recruitment to working groups difficult. One of the interviewees stressed that he/she thought that progress was sometimes slow because participants could only do what they committed to *if* they got the time and opportunity. This points to a problem related to the *empowerment and capacity of participants* to take responsibility for the network’s activities. Related to this was also the fact that representatives from primary care seldom participated in the network meetings. One of the interviewees said that this negatively affected the dynamics and “creates a hole”, making it difficult to implement changes initiated by the network, indicating that trust building was impeded due to lack of participation and progress.

Generally, it seemed important for the participants to be able to communicate practical results back to their organisation, although some of the interviewees noted a difficulty in explaining what they actually achieved at the meetings. One of the interviewees pointed out that since the meetings take three hours, they need to produce results important for the organisation as well, and not just personal satisfaction:

“It is not supposed to be only some sort of forum for personal development …you are there as a representative of your organisation and your participation should of course be beneficial to your organisation.” (Interview 1)

### Sustainability: relation building

In parallel with the activities and projects related to pragmatism was also a continuous work in line with aspects of sustainability. There was a focus on *participation* from network participants and the NAO. New participants were well received, and the NAO leaders were welcoming and encouraging. It was repeatedly mentioned in the interviews that getting to know the participants from other organisation was important (for some, this was even the primary motivation to be part of the network), and many participants by the time of the study seemed to know each other well. The observations showed that relationship building took place both formally as part of the meeting agenda and informally during breaks. For example, the meetings included sessions that aimed to increase the participants’ understanding of each other’s activities (e.g. information about new managers or changed practices) thus trying to *build capacity*. Generally, the participants expressed curiosity towards the opinions of other participants, and there was no apparent animosity, although discussion could sometimes be heated. During the breaks, the participants seemed to continue work-related conversations started at previous meetings:

“You have a coffee together, a break and you talk a little, and something happens I think. Then [when you have to contact other organisations] you know that ‘there works Lena’ whom I talked to at the meeting, and I think it becomes easier.” (Interview 2)

Nevertheless, a great deal of the relational work was carried out in the working groups tied to the network’s different projects. One of the interviewees explained that they get to know each other well by working closely together thus *building trust and consensus*.

“…you meet [in the working groups] and you create a common vision … a forum for discussion. You bring up: this is how we see it at the hospital and this is how we see it in primary care and from the municipality, and then we can meet in that and find solutions and get an increased understanding /…/ so we don’t work in opposition.” (Interview 2)

The interviewees also mentioned that they had contacted other participants between meetings to discuss, for instance, the care of specific patients or more general queries. This suggests that the meetings create new links between different organisations and *empower them* to achieve the network goal of constituting a platform (see case description).

### The role of the NAO

It was emphasized in the interviews that the NAO was hugely important for creating structure, helping concretize the participants’ ideas, and keeping them on track:

“If it wouldn’t proceed like this, then I think you would lose interest … and not come to every meeting. It is important that it is structured and that it follows a plan …”(Interview 4)

Simultaneously, the NAO leaders appeared to be flexible and perceptive, allowing time for spontaneous discussions on topics of importance for the participants. On one occasion, the NAO leaders wanted the participants to discuss what had been working well in relation to SIPs, but allowed the participants to talk about what was problematic instead when this was asked for. In general, the NAO leaders emphasised that their focus was the participants’ concerns, and to help them process ideas and get results. This suggests that the leadership style was *coordinating* and *inclusive* rather than directive, which sometimes resulted in projects developing slowly and rather far from the initial idea.

One example of this style was the discussion summarized below, centred around a workshop that the network was planning. It stretched over several meetings and gradually changed character. The workshop evolved from a one-time opportunity to establish new contacts between staff members across organisations (an idea initially formulated by the NAO), to focusing on creating a product, at the same time as basic questions such as workshop size were repeatedly discussed:

*At the September meeting, the NAO leaders bring up that the network has been granted some money to carry out an activity related to innovation. They suggest a getting-to-know-each-other-workshop to build trust. They ask whether the idea gets “a thumbs up or down”. Some participants feel that getting-to-know-each-other is too abstract and some argue that site visits or a recurring joint introductory program for new employees would be better. Someone says that it must result in ‘a product’ in order to enhance collaboration. The NAO leaders establish from the discussion that they can continue planning the workshop, and attempt to form a working group*.

*At the November meeting, it is discussed whether the workshop should be held once or twice. A network participant asks about the size and the purpose of the workshop. A few participants seem hesitant regarding how many staff members can be released for this type of activity. One of them expresses that it becomes “yet another thing that competes for the staff’s attention” and asks what happens after the workshop. A few other participants emphasize that the workshop needs to result in ‘a product’. The NAO leaders repeat that the important thing, and the purpose, is that staff members in different organisations get to know each other*.

*At the February meeting, an outline of the workshop program is presented. It is discussed again if it should be held once or twice, who it is that should come and whether managers will give their staff permission to come. One of the participants ask indignantly where it will take them if they don’t even have time for collaboration. After that, the NAO suggests that the network should start working on the subsequent product, which involves editing film material, performing interviews and creating brochures, during the autumn after the workshop that is planned to May*.

This episode from the field notes also illustrates that some decisions were made in the working groups in between meetings, and furthermore that decision-making at the meetings tended to be rather *informal*, for example when the NAO leaders asked for a “thumbs up or down”. In general, decisions during meetings were often made by the NAO leaders interpreting the participants’ opinions in a certain way, although the participants’ approval sometimes appeared rather vague. There were no formal processes for making decisions by voting or similar. While these decision-making procedures risked leaving out some views and actors, it also made it possible for the NAO to make progress, occasionally being more *directive*.

The generally inclusive leadership style however meant that the NAO leaders worked hard to *empower* the participants and try to boost the participants’ energy when it was low. For example, on one occasion, one of the meeting participants was disheartened about how few SIPs had been carried out, but one of the NAO leaders focused on small improvements described by the participant and complimented the participant and their organisation for working on improvements at all. The NAO leaders also worked hard to *include different interests* in the networks activities, and they were highly involved in trying to find members for working groups, which was sometimes problematic since all network participants had busy schedules. The NAO leaders also worked in an inclusive way and contributed to relationship building between the meetings, e.g. when they connected people and sent information to individuals within and outside of the network:

“This great climate…how shall I put it, they create that to some extent. Like, ‘have you two met?’, ‘but you work there, have you two talked?’ They help out with that, to make us meet, it is their way of leading I think”. (Interview 2)

Lastly, while initiatives and involvement from the participants were encouraged, the NAO leaders stepped in when this did not occur, making sure that the work progressed. For example, in a project that aimed to improve the situation for elderly with poor mental health, the participants agreed on mapping what types of interventions already existed and compiling the information, possibly producing a guide for senior citizens. When no one seemed motivated to do the actual work, the NAO leaders agreed to do it to advance the project, though with some hesitation since they thought it would be easier to map and compile such information via the heads of the different organisations.

## Discussion

The aim of this study was to enhance the understanding of the relationship between pragmatism and sustainability, and the role network governance plays in this respect. We found that the voluntary meso-level network studied carried out activities relating to both principles, though with an emphasis on pragmatism, i.e., on ‘getting things done’. Progress appeared essential for maintaining an interest in the collaboration among the network members. This emphasis did not seem to influence sustainability negatively, by for instance hindering inclusive and participative activities, which has been pointed out as a potential risk in previous literature. Rather, an interplay between the principles was found: Working together to execute concrete projects – not least in the smaller working groups – developed trust and consensus between the network participants.

By this, the focus on pragmatism in the network reinforced sustainability in a way that is similar to the trust-building loop described by Huxham & Vangen [13] and discussed further as the “perception of progress” mechanism by Aunger et al. [14]. One key factor for enabling trust seemed to be that the network was not taking on too demanding projects, which according to Aunger et al., [14] risks leading to failure with decreasing trust as a result. Rather, tasks or projects that were possible to carry out with existing resources and time were initiated, which simultaneously could imply a risk that the network members’ ambitions are not meet in the long term. Thus, the network’s outcome effectiveness (in contrast to process effectiveness [12]) in part depends on how effective incremental or continuous change is in contrast to radical or transformational change [21]. Vermaak et al. [21] argue that small changes are not necessarily trivial in the long run, but may accumulate into large-scale change, particularly in systems with a high level of inter-connectedness. This implies that some of the studied network’s projects could lead to more extensive changes in health and social care for the elderly. No rigorous evaluation of outcomes has, however, been made so far.

In reviewing barriers to the integration of care in interorganisational settings, Auschra [7] lists a number of barriers within the interorganisational domain (lack of leadership and coordination; missing actors; power imbalances and conflicts; differences regarding collaboration design and aims, and incompatible organisational structures). Lack of leadership and coordination was not a barrier found in our case; rather the opposite. We found at least two aspects of the NAO’s work to be important for the balance between pragmatism and sustainability. First, the NAO constituted a resource for the functioning of the network and the NAO leaders carried out many important activities that would otherwise not have been done. They also stepped in when there were no network participants available to carry out project tasks, thus facilitating the pragmatic approach and a sense of progress. This may be of particular importance in voluntary networks, where members are not obliged to take part in projects and the capacity of the network may vary over time. It may however also lead to a gradual weakening of the network. Stadtler & Karakulak [15] have in a recent study shown that NAOs and the like may “despite their good intentions” subtly weaken collaboration by replacing the members’ tasks and decision-making through so called “role drift”, concealing gaps in the collaborative process. However, in this case, we found it to be more of a temporary solution to make progress. Second, the NAO-leaders seemed to find a middle ground between the principles of pragmatism and sustainability through their leadership style, for example by reading the atmosphere during the meetings and by being open to different kinds of activities. The NAO’s leadership was generally inclusive and empowering, acknowledging differences among the participants and their organisations, focusing on coordination. However, when needed in order to move the projects forward, the leadership style could also be more directive, something that was facilitated by decision-making being rather informal. However, this strategy was used sparingly to promote the capacity of the network and its participants. The NAO performed a sort of balancing act, indicating that their leadership style counteracted the barrier of power imbalances and conflicts [7].

Furthermore, it has been suggested that tensions between core network principles tend to decrease over time [11], and since the studied network had been operating for over five years, time may be one reason why we found balance rather than tensions. For instance, an agreement on collaboration design and aims seemed to have developed over time, the lack of which is another barrier to the integration of care in interorganisational settings mentioned by Auschra [7]. However, one challenge apparent in our study was the barrier of missing actors. Primary care representatives, and in particular the health centres, some of which are private, seldom participated in the network meetings. This may create a lack of knowledge about partner organizations, which may be detrimental to the work on improving the conditions for SIPs due to the central role of primary care, though their absence may also have contributed to the broad agreement on collaboration design and aims among the network members. To some extent, the absence of primary care representatives could also indicate a problem related to the barrier of incompatible organisational structures, pointing to difficulties for primary care providers to take part in the network’s activities, although this was not explicitly investigated. It also shows that while voluntariness is sometimes perceived as beneficial for trust [14], it also comes with the difficult task of ensuring participation from all essential actors.

In sum, the network may be described as effective in terms of process since it has been running for five years, has had a constant number of participants, carries out improvement projects, and is achieving its goals according to the participants themselves [17]. Both the observations and interviews illustrate however that without the NAO, the network would have an uncertain future. From this perspective, the sustainability of the network could be questioned. Although the NAO leaders clearly worked to build capacity by empowering participants to take responsibility for actions and outcomes important to their organisations, the network is not embedded in participants’ organisations, but instead dependent on the individual participants, which change over time.

## Limitations

We investigated the activities and internal dynamics of an interorganisational network in depth over the course of one year. While one of the study’s strengths is the objectivity achieved by observations as method, it is also a resource-intensive task. As a result, due to the single case study design, the generalizability of the results is uncertain. Specific features of the network are that it is NAO-governed, operates at the meso-level, has been operating for five years, and that participation is voluntary. It also operates in an environment where the responsibility for health and social care is split between different organisations with different sources of funding [7]. We believe that our results are transferrable to similar settings with an NAO.

Another limitation is that we concentrated on the relationship between pragmatism and sustainability, and did not include other principles such as flexibility and accountability [9]. The two studied principles, and the role of network governance for their interdependence, are however currently at issue in the contemporary literature, and are considered highly relevant when investigating the effectiveness of networks [11,14]. Thus, while the complexity of network functioning is not fully acknowledged, given the study aim of examining the relationship between this particular set of principles in detail, the limitation was deemed acceptable.

## Conclusion

The emphasis on pragmatism we found in the observations and interviews did not seem to impact the sustainability of the network negatively; joint work on projects and the perception of progress rather reinforced aspects of sustainability such as trust building and consensus. The role of the NAO appeared to be crucial for ensuring progress, for example by constituting a resource in the projects, which may be linked to the network being voluntary. The balancing of the two principles by switching from a coordinating leadership style to a more directing one when necessary suggests that governance and different leadership styles should be carefully considered when initiating interorganisational networks for integrated care. We suggest that future studies further explore how aspects of network functioning relates to network outcomes in order to capture the real-world value of interorganisational networks. Furthermore, a focus on the importance of participation being voluntary rather than mandatory for network functioning, and how effective interorganisational networks are in contexts with a large share of private actors in both the healthcare and social care sectors, is warranted.
